# The Role of Hypoxia-Induced Mitogenic Factor in Organ-Specific Inflammation in the Lung and Liver: Key Concepts and Gaps in Knowledge Regarding Molecular Mechanisms of Acute or Immune-Mediated Liver Injury

**DOI:** 10.3390/ijms22052717

**Published:** 2021-03-08

**Authors:** Sananda Pai, Dolores B. Njoku

**Affiliations:** 1 Department of Anesthesiology and Critical Care Medicine, Johns Hopkins University, Baltimore, MD 21287, USA; spai9@jhu.edu; 2 Department of Pediatrics, Johns Hopkins University, Baltimore, MD 21287, USA; 3 Department of Pathology, Johns Hopkins University, Baltimore, MD 21287, USA

**Keywords:** HIMF, RELM-a, RELM-b, FIZZ1, retnla, liver

## Abstract

Hypoxia-induced mitogenic factor (HIMF), which is also known as resistin-like molecule α (RELM-α), found in inflammatory zone 1 (FIZZ1), or resistin-like alpha (retlna), is a cysteine-rich secretory protein and cytokine. HIMF has been investigated in the lung as a mediator of pulmonary fibrosis, inflammation and as a marker for alternatively activated macrophages. Although these macrophages have been found to have a role in acute liver injury and acetaminophen toxicity, few studies have investigated the role of HIMF in acute or immune-mediated liver injury. The aim of this focused review is to analyze the literature and examine the effects of HIMF and its human homolog in organ-specific inflammation in the lung and liver. We followed the guidelines set by PRISMA in constructing this review. The relevant checklist items from PRISMA were included. Items related to meta-analysis were excluded because there were no randomized controlled clinical trials. We found that HIMF was increased in most models of acute liver injury and reduced damage from acetaminophen-induced liver injury. We also found strong evidence for HIMF as a marker for alternatively activated macrophages. Our overall risk of bias assessment of all studies included revealed that 80% of manuscripts demonstrated some concerns in the randomization process. We also demonstrated some concerns (54.1%) and high risk (45.9%) of bias in the selection of the reported results. The need for randomization and reduction of bias in the reported results was similarly detected in the studies that focused on HIMF and the liver. In conclusion, we propose that HIMF could be utilized as a marker for M2 macrophages in immune-mediated liver injury. However, we also detected the need for randomized clinical trials and additional experimental and human prospective studies in order to fully comprehend the role of HIMF in acute or immune-mediated liver injury.

## 1. Introduction

Hypoxia-induced mitogenic factor (HIMF) is a cysteine-rich secretory protein and cytokine [1]. HIMF is also known as resistin-like molecule α (RELM-α), found in inflammatory zone 1 (FIZZ1), or resistin-like alpha (retlna). HIMF has mitogenic and chemotactic properties and contributes to inflammation, angiogenesis, and fibrosis [2,3]. Under homeostatic conditions, HIMF is found in many tissues, including the mammary tissues, heart, spleen, muscle, white adipose tissue, bladder, vascular endothelial tissues, lung, and liver; however, its function in most tissues is not well defined [1]. This lack of characterization is unfortunate since HIMF may have critical functions in the liver that have yet to be defined.

Over 20 years ago, a study described a protein with the initials HIMF that referred to hyperimmune milk factor [4]. This protein had anti-inflammatory properties and was believed to act through the reduction of tight junction permeability and movement of inflammatory cells [5]. Given that hyperimmune milk factor and hypoxia-induced mitogenic factor both share the same initials, play a role in chemotaxis and inflammation, and are found in the mammary glands, this might suggest that they are the same molecule. Along these lines, an earlier study also described HIMF as a hormone with high concentrations in fat cells in the mammary gland; however, the function of HIMF was poorly defined [6].

The first instance of the modern version of HIMF/FIZZ1 was described in 2000 when researchers discovered a 9.4-kDA protein in the bronchiolar lavage fluid using an allergic pulmonary inflammation mouse model. This protein belonged to the newly discovered *FIZZ* gene family and therefore was named FIZZ1 [1]. Other researchers simultaneously discovered the same gene family while searching for homologs of resistin. However, they called this gene family RELM to denote its similarity to the resistin molecule [6]. In both of these studies, RELM-beta/FIZZ2, another member of the gene family, was also discovered in mice. It was later determined that there were differences in RELM protein expression in mice and humans. Hence, RELM-alpha, RELM-beta, and RELM-gamma are present in mice, while the only member of the *RELM* gene family in humans is RELM-beta. Subsequently, RELM-beta was established as the human homolog of HIMF [7]. Thus, for the purposes of this review, HIMF refers to RELM-alpha in mice and RELM-beta in humans.

The majority of papers discussing the role of HIMF demonstrate its functions in the lung, where it is primarily produced by endothelial cells. HIMF directly induces cell proliferation that causes vascular remodeling [8]. When HIMF was administered into the lungs of rats, the rats developed pulmonary hypertension. A subsequent manuscript describing roles for HIMF (FIZZ1) found that pulmonary fibrosis was a result of HIMF-regulated Th2-mediated mechanisms likely via IL-4 an IL-13 since HIMF expression was significantly decreased in IL-4 and IL-13 knockout mice [9].

Other mechanisms have been attributed to HIMF-mediated pulmonary fibrosis. A prior manuscript also found that HIMF activates phosphoinositide-3 kinase (PI3K)/Akt and ERK (p42/p44 MAP kinase)-dependent pathways that promote pulmonary smooth muscle and endothelial cell proliferation [2,10]. In humans, HIMF (RELM-beta) led to airway remodeling via fibroblast proliferation and differentiation [11]. Furthermore, in asthmatic patients, HIMF (RELM-beta) increased smooth muscle proliferation and induced vascular remodeling using this same signaling pathway demonstrated in mice [12]. HIMF is also expressed in lung bone-marrow-derived hematopoietic progenitor cells; however, its function in these cells is not completely clear.

While large numbers of papers have investigated roles for HIMF in the lung, there are fewer studies investigating HIMF in other organs. There are no papers investigating HIMF in the spleen, in spite of its effects on immune cells. Regarding the white adipose tissue, HIMF was found along with resistin, but the role was unclear. However, in the muscle, HIMF is prevalent in inflammatory myopathy. In this disease, HIMF upregulates IL-18 in myoblasts, which acts as a pro-inflammatory cytokine and regulates calcium mobilization in smooth muscle cells [13,14]. Similar to lungs, HIMF activates IL-18 pathways through PI3K/Akt pathways, which promotes angiogenesis [15]. In the same way, in the heart, HIMF seems to have an inflammatory role where it promotes cardiac fibrosis using IL-6 as a downstream signal. HIMF also plays a role in cardiomyocyte hypertrophy and myocardial fibrosis, which acts through the MAPK and CAMKII-STAT3 pathways [16]. In addition, HIMF (FIZZ1) promotes angiogenesis in rat aortic endothelial cells through upregulation of Gng8 and Atg9a. In sharp contrast, in the gut, HIMF is thought to have cancer properties because RELM-beta was upregulated in the colon of colon cancer patients [17]. Mouse and human RELMβ were shown to have bactericidal activity in the colon and promote host–bacteria mutualism in the microbiota of the intestine [18]. Although its role in cancer is still being clarified, HIMF (RELM-beta) is highly expressed in a human colon cancer cell line, LIS174T, providing systems that could be utilized in future studies [19].

Conflicting roles for HIMF have been found in diseases affecting the liver. HIMF promotes the repair of the liver and reduces liver toxicity from acetaminophen intoxication [20]. In sharp contrast, HIMF (RELM-beta) exacerbates the severity of nonalcoholic steatohepatitis [21]. In the liver, eosinophils are the primary producers of HIMF [22]. A prior study found that HIMF (FIZZ1) correlates with IL-21 expression and increased severity of liver granulomas [23]. HIMF also upregulates the expression of fetal-liver kinase 1, which promotes endothelial cell proliferation and survival, via the PI3K/Akt-NF-κB signaling pathway [24].

Although a large number of studies describe the actions of HIMF in the lung, very few studies focus on the role of HIMF in the liver and how hepatic and pulmonary mechanisms might be connected. The majority of studies show that eosinophils are primary producers of HIMF (Retnla) in the liver, while epithelial cells were primary producers of HIMF (Retnla) in the lung. However, multiple studies utilize HIMF in both the liver and the lung as a marker for alternatively activated macrophages. M2, also known as alternatively activated macrophages, have protective or pathogenic roles in liver injury. This duality in the function of HIMF suggests to us that previously unrecognized mechanisms could have relevance in understanding liver injury from drugs. To this point, there have been no comprehensive reviews that dissect the importance of HIMF in both the lung and the liver. This is important because HIMF appears to have differential organ-specific effects that are not appreciated when its role in a single organ is assessed. This means to us that therapies and practice guidelines designed to address HIMF in one organ may have positive or negative effects in another. Immune-mediated liver injury is a common hepatic drug-induced hypersensitization process that can lead to the removal of approved medications from the commercial market. However, underlying mechanisms remain poorly understood. The aim of this focused systematic review is to analyze the literature and examine the effects of HIMF and its human homolog in organ-specific inflammation in the lung and liver. We will evaluate research published from 1955 to 2021. Our objective is to decipher the literature about HIMF in the lung and liver to identify key concepts and potential gaps in knowledge regarding molecular mechanisms of acute or immune-mediated liver injury. Furthermore, we would like to promote the research of HIMF in the liver to assess its therapeutic potential in acute or immune-mediated liver injury.

## 2. Methods

### 2.1. PRISMA Criteria

We followed the guidelines set by PRISMA (Preferred reporting items for systematic reviews and meta-analyses) in constructing this review [25]. The relevant checklist items from PRISMA were included. Items related to meta-analysis were excluded.

### 2.2. Protocol and Registration

This protocol includes a focused systematic review of health research studies including human subjects and mouse subjects. We will register this protocol once peer review has been completed if necessary.

### 2.3. Eligibility Criteria

Our eligibility criteria included health research, as well as mouse research, involving HIMF and organ-specific inflammation in the lung and liver. This search was from 1955 to 2021. Human studies that were considered included randomized control trials, clinical studies, comparative studies, observational studies, reviews, systematic reviews, and controlled clinical trials. Mouse research had to have direct relevance to human health. The observed outcomes were inflammatory or regulatory effects of HIMF in the lung and liver.

### 2.4. Information Sources

Studies were identified by searching PubMed and Ovid and browsing reference lists of selected articles. Limitations were placed on language, including only articles published in English. The last search was run on 23 January 2021.

### 2.5. Search

Relevant keywords were searched in various combinations. The keywords included, but were not limited to: HIMF, RELM-a, FIZZ1, Retnla, RELM-b, lung, liver, proinflammatory, inflammatory, protection, and pathogenesis. Table 1 provides a more detailed list of the keywords used in the search strategy and inclusion criteria in terms of population of interest, intervention, comparison group, and outcome. Searches involved the following terms: HIMF AND mouse, HIMF AND rat, HIMF AND lung, HIMF AND human, HIMF AND patient, HIMF AND liver, HIMF AND lung, RELM-a AND mouse, RELM-a AND rat, RELM-a AND lung, RELM-a AND human, RELM-a AND patient, RELM-a AND liver, RELM-a AND lung, FIZZ1 AND mouse, FIZZ1 AND rat, FIZZ1 AND lung, FIZZ1 AND human, FIZZ1 AND patient, FIZZ1 AND liver, FIZZ AND lung, Retnla AND mouse, Retnla AND rat, Retnla AND lung, Retnla AND human, Retnla AND patient, Retnla AND liver, Retnla AND lung RELM-b AND mouse, RELM-b AND rat, RELM-b AND lung, RELM-b AND human, RELM-b AND patient, RELM-b AND liver, RELM-b AND lung. The search was not limited by date of publication but was limited by inclusion of both HIMF and the organ system in question.

### 2.6. Study Selection

**Figure 1:**
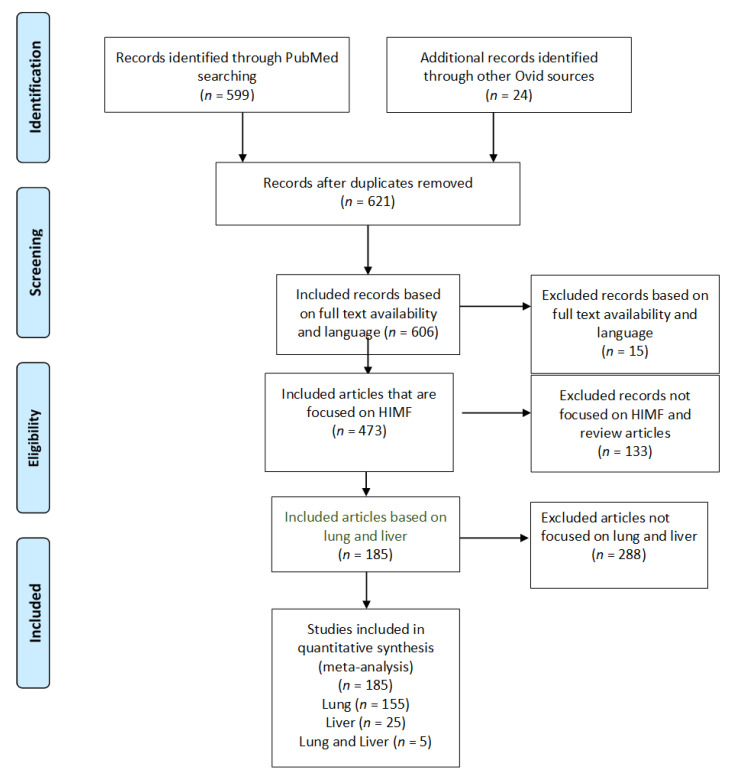
PRISMA study selection flow diagram. The PRISMA flow diagram demonstrates each phase of the search strategy in which articles were evaluated. During each phase, articles were excluded based on our defined criteria. The last phase of exclusion resulted in a final collection of articles, which were then divided by organ systems.

The study selection is indicated in Figure 1 by the PRISMA flow diagram [25]. An electronic database search of PubMed with several key terms in various permutations was performed. The articles were evaluated and articles that did not meet inclusion criteria were excluded. During this stage, articles were included if the full-text article elaborated on the information included in the abstract to clarify the population and comparison. Case reports were excluded due to the reduced level of generalizability. This stage evaluated the intervention based on full-text searches. All articles not discussing the desired interventions, such as HIMF, regardless of the name, were excluded. The next stage removed additional articles not meeting inclusion criteria. The desired comparison intervention was the specific organ systems of interest (hepatic, pulmonary), as well as the comparison between murine and human models.

### 2.7. Data Collection Process

Information was extracted from each of the selected studies to provide summative evidence on the protective or pathogenic effects of HIMF within each organ system, as well as between human and mouse models (C57BL/6 and BALB/c).

### 2.8. Data Items

Information was extracted for each included study on: (1) the protective effects of HIMF; (2) the pathogenic effect of HIMF; (3) the inflammatory effects of HIMF; (4) the organ-specific effects, including in the liver and pulmonary system.

### 2.9. Risk of Bias in Individual Studies

The Cochrane RoB 2.0 tool is commonly used for meta-analysis of randomized clinical trials. This tool was adapted to perform a literature analysis. The risk of bias was assessed in individual studies using the Cochrane RoB 2.0 tool. We analyzed the collected data for selection bias, confirmation bias, publishing bias, observation bias, and reporting bias. We assessed the selection of the reported results, measurement of the stated outcomes, missing outcomes data, deviation from the intended result, and the randomization process. Selection in the studies analyzed was defined as the identification of a group of data for analysis in a way that interfered with randomization and proper representation of the study population. The Cochrane RoB 2.0 tool was used to evaluate selection bias by assessing whether the allocation of data was random and whether any baseline difference between groups could suggest a problem with randomization. Confirmation bias in the analyzed studies was defined as a tendency to interpret information or results in a way that confirmed prior preconceptions. An example of confirmation bias would be a manuscript that only acknowledges pulmonary roles for HIMF, while the results could also represent hepatic roles for this protein. The Cochrane RoB 2.0 tool was used to evaluate confirmation bias by searching for deviations from intended goals stated in the aims of each article that could be demonstrated by the interpretation of the data in a way that confirmed prior preconceptions. Publication bias was defined as when the outcome of a study influenced the decision regarding whether to publish it. An example could be the wealth of data on pulmonary but not hepatic roles for HIMF. The Cochrane RoB 2.0 tool was used to evaluate publication bias by evaluating the potential presence of missing outcome data that could represent an alternative conclusion. Observation bias was defined as the tendency to see what we expect to see. The Cochrane RoB 2.0 tool was used to evaluate observation bias by assessing the measurements used to determine the outcome and whether the measurements were appropriate, and could have differed between groups. An example of observation bias would be a manuscript that only focused on the pulmonary roles of HIMF and did not include the hepatic roles, although the evidence might suggest that they were present. Reporting bias was defined as selective reporting of some outcomes that appeared to depend on the nature and direction of the results.

### 2.10. Summary Measures

The primary outcomes of interest were proinflammatory, inflammatory, protection, or pathogenesis effects in organ-specific inflammation of the lung and liver. Risk ratios and differences between the means were not analyzed.

### 2.11. Synthesis of Results

Results were synthesized based on the measures reported in descriptive and mechanistic studies. Descriptive studies included the intervention and a detailed account of the outcome. Mechanistic studies involved using the intervention to explore mechanisms of action, as well as adding a treatment to examine the effects of the treatment on the intervention. Mechanistic studies were our goal in which HIMF was investigated in vitro or in vivo; however, our preference was in vivo.

### 2.12. Risk of Bias across Studies

The risk of bias was assessed across studies using the Cochrane RoB 2.0 tool. Similar parameters were used to assess risk of bias across studies as in individual studies.

### 2.13. Additional Analyses

No additional analyses were performed.

## 3. Results

### 3.1. Study Selection

Utilizing our inclusion and exclusion criteria, 606 studies were deemed eligible for assessment (Figure 1). All case reports were excluded. Of these articles, 133 were excluded because they did not explicitly mention HIMF or because they were review articles. Further, 473 articles were deemed appropriate for further analysis. Of these, 288 articles were removed because they did not focus on the pulmonary or hepatic systems. We identified 185 full-text articles that included information about HIMF at an organ-specific level. Of these 185 articles, 155 included information about HIMF in the pulmonary system, 25 included information about HIMF in the liver, and 5 included information about HIMF in both the pulmonary system and the liver. Among the 155 articles about HIMF in the pulmonary system, 6 utilized human subjects, 142 utilized a murine model, and 7 utilized both human and murine models. Within the articles discussing HIMF in the liver, all 25 utilized murine models. Finally, within the articles discussing HIMF in both the pulmonary system and liver, all five utilized murine models.

### 3.2. Effects of HIMF on the Pulmonary System

#### 3.2.1. Human Subjects—Pulmonary Hypertension and Pulmonary Fibrosis

The primary area in which HIMF has been investigated in human subjects is in the pathogenesis of pulmonary hypertension and pulmonary fibrosis. HIMF (RELM-beta) is upregulated during hypoxia in the smooth muscle cells of pulmonary arteries, causing smooth muscle cell proliferation that contributes to pulmonary hypertension [26]. Additionally, when compared to normal controls, patients with scleroderma-associated pulmonary hypertension have increased HIMF (RELM-beta) levels in the endothelium, vascular smooth muscle cells, plexiform lesions, macrophages, T cells, and myofibroblast-like cells [27]. Along these same lines, HIMF (RELM-beta) levels were increased in the lungs of patients with idiopathic pulmonary fibrosis when compared to normal controls [28]. Furthermore, in idiopathic pulmonary hypertension, HIMF stimulates autophagy and bone morphogenetic protein receptor type 2 (BMPR2) impairment. This impairment is thought to occur via the high mobility group box protein-1 (HMGB-1)/receptor for advanced glycation endproducts (RAGE)-mediated pathway in endothelial and smooth muscle cells of the pulmonary arteries [29]. Finally, similar to a previously mentioned study, HIMF was upregulated in the macrophage-like inflammatory cells in patients with idiopathic pulmonary hypertension [30]. However, whether or not HIMF has similar effects on endothelial cells in the liver in acute or immune-mediated liver injury is not completely clear.

#### 3.2.2. Human Subjects—Asthmatic/Allergic Inflammation

The second area in which HIMF has been strongly investigated is asthmatic and allergic inflammation. In a study that measured the pulmonary HIMF (FIZZ1) levels as a marker for alternative macrophage activation in asthmatic patients, it was found that HIMF levels were similar in asthmatic and normal control patients [31]. However, other studies found that HIMF (RELM-beta) was increased in the bronchial mucosa of asthmatic patients when compared to healthy patients [11,12,32]. Although these studies did not affect these topics, differences in outcome may have been affected by age, sex, and the severity of the disease. Thus, questions remain regarding the role of HIMF in pulmonary disease seen in patients. To better understand the mechanisms of this disease, we reviewed mouse studies regarding pulmonary fibrosis and allergic asthma.

#### 3.2.3. Murine Model—Pulmonary Hypertension

HIMF is thought to play an important role in pulmonary hypertension and associated pulmonary vascular remodeling. Increased levels of HIMF have been found in the bronchoalveolar lavage fluids of mice with pulmonary hypertension [33]. HIMF led to an increase in perivascular and peribronchial collagen deposition, and these effects were decreased in HIMF-deficient mice [34]. This HIMF (RELM-alpha) expression contributes to pulmonary arterial remodeling and is upregulated by Th2 and IL-13-mediated inflammation [35]. Blocking HIMF expression also blocked increases in mean pulmonary arterial pressure, pulmonary vascular resistance, and vascular remodeling caused by hypoxia, showing that HIMF functions as a cytokine-like factor in these processes [36]. Furthermore, during the pulmonary hypertension remodeling process, HIMF stimulates the recruitment of bone-marrow-derived mesenchymal stem cells for remodeling of the pulmonary vasculature [37]. In pulmonary arterial hypertension, S100A1 mediates the HIMF-induced smooth muscle cell migration, vesicular exocytosis, and nuclear activation [14]. Furthermore, HIMF levels are increased in airway epithelial and inflammatory cells in chronic hypoxia or antigen sensitization but are only increased in pulmonary vasculature tissue in chronic hypoxia [37]. HIMF also contributes to vascular inflammation by inducing endothelial cell apoptosis in the lung during pulmonary hypertension [38].

The PI3K/Akt pathway has been implicated in many pulmonary hypertension studies utilizing a murine model. In a chronic hypoxia model of pulmonary hypertension, HIMF was upregulated by hypoxia and caused proliferative effects through the PI3K/Akt pathway [2]. In particular, HIMF enhances vascular endothelial growth factor (VEGF) and fetal liver kinase-1 (Flk-1) production in mouse lung epithelial cells in a PI3K/Akt-NF-κB signaling pathway-dependent manner, as seen in a murine model with intratracheally instilled recombinant HIMF [24]. Similarly, intravenous injection of HIMF in mice increased CD68-positive inflammatory cells in the lung and caused VEGFR2 downregulation, showing that HIMF facilitates the pulmonary inflammation and angiogenesis that is often seen in pulmonary hypertension using VEGF [10].

Another possible mechanism for the pulmonary-hypertension-promoting abilities of HIMF has been found: HIMF increases intracellular calcium levels in the human pulmonary artery smooth muscle cells via the PLC signaling pathway in an IP3 and tyrosine phosphorylation-dependent manner, which ultimately leads to constriction of the pulmonary vasculature [13]. Furthermore, binding of HIMF with calcium-sensing receptor stimulates pulmonary hypertension [38].

The roles of IL-4 and STAT6 are also critical to HIMF-mediated pulmonary hypertension. In a hypoxia-induced pulmonary hypertension model, Th2 regulation did not play a role since IL-4 and STAT6-deficient mice had the same HIMF expression levels as wild-type mice. However, although the HIMF expression levels were similar, the IL-4 and STAT6-deficient mice had decreased levels of HIMF-induced proliferative activity and chemokines than the wild-type mice, suggesting a significant role of IL-4 in HIMF-induced lung inflammation [39]. Finally, HIMF can also induce hypoxia inducible factor-1 (HIF-1), VEGF-A, and IL-6, which can stimulate hypoxic inflammation and pulmonary hypertension [40]. IL-4 and STAT6 promote immune-mediated liver injury; however, the role of HIMF in this type of liver injury is essentially unknown [41].

#### 3.2.4. Murine Model—Pulmonary Fibrosis

In contrast to human studies, there have been many studies investigating pulmonary fibrosis using validated mouse models. In bleomycin-induced pulmonary fibrosis, HIMF (FIZZ1) expression was found in the alveolar and airway epithelium, but not in isolated lung fibroblasts. In particular, type II alveolar epithelial cells expressed HIMF (FIZZ) and stimulated alpha smooth muscle actin and type 1 collagen expression [42]. These authors determined that the HIMF (FIZZ1) expression that occurs in the type II alveolar epithelial cells is induced by IL-4 and IL-13 stimulation through STAT6, which ultimately leads to fibrosis. Other signaling molecules, such as Notch1, have been implicated in HIMF-induced fibrosis. Liu et al. demonstrated that HIMF induced Hes1, which is involved in increasing alpha-smooth muscle actin expression levels, via Notch1 and its ligand Jagged1 [43]. Lastly, bleomycin-treated HIMF (FIZZ1)-deficient mice have reduced levels of pulmonary fibrosis and HIMF (FIZZ1) overexpression exacerbates fibrosis, portraying the profibrogenic role of HIMF [9].

On the contrary, HIMF recruited bone-marrow-derived cells to the lung; however, it did not increase airway inflammation or fibrosis independently in the lung [44]. Bleomycin-treated paired immunoglobulin-like receptor B (PirB)-deficient murine macrophages have increased levels of profibrogenic HIMF (RELM-alpha), suggesting that HIMF may have a B-cell-activation-independent role in fibrogenesis [45].

#### 3.2.5. Murine Model—Asthmatic/Allergic Inflammation

HIMF expression is increased during allergic pulmonary inflammation [1]. HIMF (FIZZ1) was discovered in the alveolar epithelial cells in mice facing allergic pulmonary challenge, and this upregulated expression caused type-1 and alpha-smooth muscle cell proliferation [46]. The importance of HIMF in asthma is solidified given that HIMF (FIZZ1) has been identified as a biomarker of asthma and oxidative stress [47,48]. Increased airway hyperresponsiveness is correlated with increased levels of HIMF (FIZZ1) [49]. Furthermore, the fungus, *Alternaria*, which is associated with severe asthma, increases HIMF expression, thereby promoting airway fibrosis and epithelial thickness [50].

Interestingly, mice that overexpressed HIMF (retnla) had decreased levels of inflammatory cells when challenged with ovalbumin [51]. However, HIMF-deficient (retnla) mice had decreased amounts of muscle vascularization and perivascular inflammation when induced by ovalbumin [11]. Similarly, Lunasin, which decreases the amount of HIMF expression in the lung after ovalbumin challenge, also decreased inflammation. In *Aspergillus*-induced allergic airway disease, HIMF (RELM-beta) deficiency led to decreased tissue inflammation and less markers of chronic disease [52]. Finally, B cell activating factor deficiency decreased the HIMF expression levels in OVA-induced allergic inflammation mice [53]. Thus, although one paper has suggested that HIMF overexpression decreased inflammation, the majority of papers suggest that HIMF has a proinflammatory role.

There are multiple studies that have provided information about some of the pathways and molecules involved in the role of HIMF in allergic inflammation. In particular, STAT6 and CCAAT-enhancer binding protein (C/EBP) regulate the IL-4 and IL-13-induced HIMF expression that occurs in this type of allergic inflammation [54]. Similarly, reduced HIMF levels due to the absence of IL-4 and STAT6 correlated with decreased lung inflammation and eosinophil levels [55]. Additional studies have shown that VCAM-1 upregulation by HIMF through the PI3K/Akt pathway also induced IkB kinase, which led to NF-κB activation [24]. Furthermore, HIMF (FIZZ1) was increased in an allergic asthma mouse model and was positively correlated with VEGF levels and percentage of vascularity [56]. Transgenic mice that are positive for BMPR2 expressed lower levels of HIMF (FIZZ1) than WT mice following ovalbumin-induced inflammation, suggesting that the *BMP2* gene may also have a role in HIMF signaling pathways [57]. Finally, HIMF (FIZZ1) is correlated with PTEN inhibition, which leads to the increased expression of type-1 collagen and fibronectin-1 in airway remodeling asthma [58]. This evidence shows that PI3K/Akt pathways have a major role in HIMF signaling, but other molecules may also be involved.

Interestingly, mice exposed to cigarette smoke had increased levels of HIMF (FIZZ1) and increased pulmonary inflammation, but both are reduced after exercise training [59]. Another carcinogen, NNK, also increased the HIMF (retnla) expression levels and inflammatory lung response [60].

#### 3.2.6. Murine Model—Parasitic Infection

Because HIMF is believed to have a significant role in Th2-type inflammation, HIMF has been utilized to investigate the pathogenesis of parasitic infection. HIMF is thought to be protective in murine models of parasitic infection. HIMF upregulation after parasitic implantation surgery does not require B or T cells, but does require IL-4 and IL-13 [61]. After *S. mansoni* infection, HIMF (RELM-alpha)-deficient mice have shown increased Th2 cytokine-dependent lung inflammation, increased fibrosis, and increased pulmonary vascularization compared to wild-type mice [62]. In *Schistosoma-*infected mice, HIMF (retnla) controlled the magnitude of the inflammatory response and promoted host survival [63]. Impaired HIMF (RELM-beta) levels as a result of IL-13 deficiency in hookworm-infected mice also caused an impairment of eosinophil expression [64]. Lung eosinophils can also be activated by IL-33, which can lead to the activation of HIMF independently of IL-4 and cause inflammation [65].

In *N. brasiliensis*-affected mice, there was an inverse correlation between disease severity and IL-13 and HIMF (retnla) [66]. HIMF (RELM-alpha) was required to decrease parasitic *N. brasiliensis* immune responses, which suggests its role as an immune brake by limiting tissue damage in the lung [67]. After *N. brasiliensis* infection, HIMF (RELM-alpha) was stimulated by Ym1 and led to tissue repair in the lung [68]. HIMF (RELM-alpha) was also required for protection from fatal lung damage and these protective effects disappeared in HIMF-deficient mice [69]. These studies suggest that HIMF might have a protective role in the lung after parasitic infection. Interestingly, schistosomiasis affects the liver, and recovery from acute and immune-mediated liver injury requires liver repair, which suggests that similar mechanisms may be at play in these forms of liver injury.

#### 3.2.7. Murine Model—Other Roles/Information Not Previously Described

HIMF has been shown to reduce apoptosis in embryonic murine lungs and is temporally and spatially co-expressed with HIF-2, suggesting a possible regulatory function of HIMF in lung maturation [70]. Similarly, after murine pneumonectomy, HIMF is upregulated in compensatory lung growth and stimulates cell proliferation, which suggests that HIMF may have a regulatory role as a lung-specific growth factor [71]. Additionally, HIMF also stimulates Surfactant B (SP-B) and C (SP-C) expression by preventing SP-B and SP-C mRNA degradation via the PI3K/Akt and ERK 1/2 pathways, suggesting another regulatory role for HIMF in lung development [72]. HIMF (FIZZ1) has been shown to have anti-apoptotic effects through the inhibition of caspase-3 and caspase-8 [73]. Additionally, Ets-1 promotes HIMF expression in developing mouse lungs, further showing HIMF’s involvement in regulating the pulmonary maturation process [74]. Finally, HIMF (RELM-alpha) also increases bone-marrow-derived stem cell proliferation in the lung without altering the differentiation potential, which provides further evidence for a regulatory role for HIMF in lung development [75].

In LPS-induced lung injury, HIMF was upregulated and stimulated VCAM-1 expression and mononuclear cell sequestration, while preventing an LPS-induced Surfactant C decrease and cell death of alveolar cells [76]. In acute lung injury, bone marrow mesenchymal stem cells improve lung injury independently of HIMF, since HIMF-expressing mesenchymal stem cells increased lung edema and leukocyte infiltration [77]. To summarize, HIMF action in the lung primarily settles around Th2 inflammation via IL-4 and IL-13 and these mechanisms are responsible for the induction of immune-mediated liver injury. However, whether or not HIMF or Th2 inflammation in the lung has a similar role in immune-mediated liver injury is unknown.

The majority of the data regarding HIMF in the pulmonary system suggests that HIMF is upregulated in pulmonary fibrosis, pulmonary hypertension, and allergic asthma, and stimulates cell proliferation and pulmonary vascular remodeling. HIMF stimulates these processes by using IL-4, IL-13, and STAT6 as key mediators, in addition to the PI3K/Akt pathway. However, there is evidence for HIMF having protective roles in models of parasitic infection, which shows that HIMF may have disease-specific effects.

### 3.3. Effects of HIMF on the Liver

#### 3.3.1. Murine Model—Parasitic Infection

HIMF is also known to have a role in parasitic infection in the liver. Increased levels of HIMF (FIZZ1) provided evidence for alternative macrophage activation after *F. hepatica* liver infection [78]. However, in the liver granulomas of IL-5-deficient mice, IL-13 decreased HIMF expression after *S. mansoni* infection, suggesting an IL-13 effector function [79]. Similarly, in a liver granuloma model of *S. mansoni* infection with IL-21-deficient mice, there was reduced IL-4 and IL-13, consequently resulting in decreased HIMF (FIZZ1) levels as well [23]. Furthermore, HIMF (FIZZ1) expression was increased in the macrophages from Th2-polarized mice with liver granulomas when compared to unpolarized mice with liver granulomas induced by *S. mansoni* infection [80].

On the contrary, the absence of HIMF (retnla) in both the lung and the liver caused increased granuloma formation after *S. mansoni* infection in both of these organs. Similarly, the absence of HIMF (retnla) also caused increased fibrosis and hepatosplenic disease in the liver and these results show that HIMF (retnla) is a positive regulator of Th2 responses in both the lung and the liver [22].

HIMF (FIZZ1) expression levels in the liver are also positively correlated with the levels of T-bet in a murine model of *S. mansoni* infection [81]. In the liver, after *F. hepatica* infection, the lack of MyD88 had no effect on HIMF (RELM-alpha) expression levels, showing that toll-like receptors (TLRs) are not involved in parasite-induced liver inflammation [82].

*S. mansoni* has been shown to regulate alternative macrophage activation by decreasing HIMF (retnla) expression, which controls the granuloma formation in the liver in response to the infection [83]. Fasciola hepatica tegumental antigens (FhTeg) can induce HIMF independently of IL-13 [84]. During *F. hepatica* infection in the liver, cells that have intermediate levels of expression of heme oxygenase-1 (HO-1) have high levels of HIMF (FIZZ1) expression, suggesting alternative macrophage activation [85].

#### 3.3.2. Murine Models—Liver Injury

Very few papers have assessed the role of HIMF in models of liver injury. We did not discover evidence for the induction of HIMF in acetaminophen-induced liver injury using wild-type mice. However, HIMF was induced in Galectin-3 KO mice following acetaminophen treatment, and it was suggested that HIMF partially contributed to protection in this study. [20]. Elevated levels of HIMF (FIZZ1) were detected in fatty acid binding protein-5 (FABP-5)-deficient mice that demonstrate resistance to LPS-induced immune-mediated liver [86]. Conversely, quaking protein 1 deficiency, which has been shown to downregulate levels of HIMF (FIZZ1), worsened LPS-induced liver injury in mice [87]. In rats with nonalcoholic steatohepatitis, an anti-inflammatory phenotype correlated with increased markers for alternatively activated macrophages, including HIMF (retnla) [88]. Conversely, another study found that HIMF (RELM-beta) is required in both the liver and the gut for the development of nonalcoholic steatohepatitis [21].

Thus, similar to the lung, studies in the liver suggest that HIMF likely has protective and pathogenic effects. HIMF appears to offer protection from acute liver injury and appears to reduce damage from immune-mediated liver injury. Next, we needed to analyze the quality of data using the PRSMA risk of bias assessment tool.

### 3.4. Risk of Bias Assessments

#### 3.4.1. Overall Risk of Bias Assessment for ALL Articles

**Figure 2:**
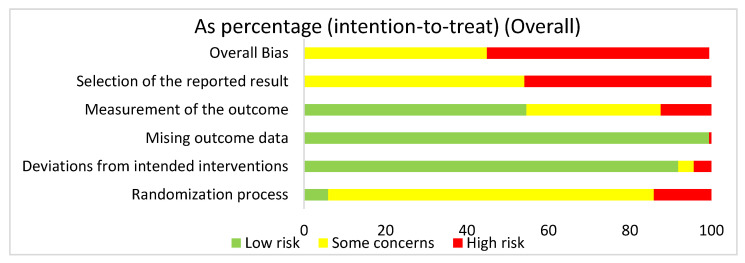
The overall risk of bias for all the articles in the dataset was a high risk of bias (54.6%) and some concerns (44.8%). The selection of the reported result had a high risk of bias (45.9%) and some concerns (54.1%). The measurement of the outcome had a high risk of bias (12.4%), some concerns (33%), and low risk of bias (54.6%). The missing outcome data domain had a low risk of bias (99.5%) and a high risk of bias (0.5%). Deviations from intended interventions had a low risk of bias (91.9%), some concerns (3.8%), and high risk of bias (4.3%). The randomization process had a high risk of bias (14.1%), some concerns (80%), and low risk of bias (5.9%).

We used the risk of bias tool even though we were analyzing both human and mouse studies. There were no randomized control trials. We selected intention to treat in order to detect inadvertent reporting or analysis bias in our studies. In order to determine the overall risk of bias, we assessed the selection of the reported results, measurement of the stated outcomes, missing outcome data, deviation from the intended result, and the randomization process. In assessing the risk of bias in studies regarding HIMF and either the pulmonary system or liver, we discovered that overall risk of bias for all human and mouse studies exposed some concerns (44.9%) or high risk of bias (55.1%) (Figure 2). The greatest contributor to the overall risk of bias seemed to be the selection of the reported result domain, which had some concerns (54.1%) or a high risk of bias (45.9%). In contrast, the measurement of the outcome was primarily low risk of bias (54.6%). Missing outcome data and deviations from the intended interventions also yielded primarily a low risk of bias of 99.5% and 91.9%, respectively. Not surprisingly, when looking at the overall bias, the randomization process primarily showed some concerns (80%) or high risk of bias (14.1%).

#### 3.4.2. Lung: Overall Risk of Bias Assessment

**Figure 3:**
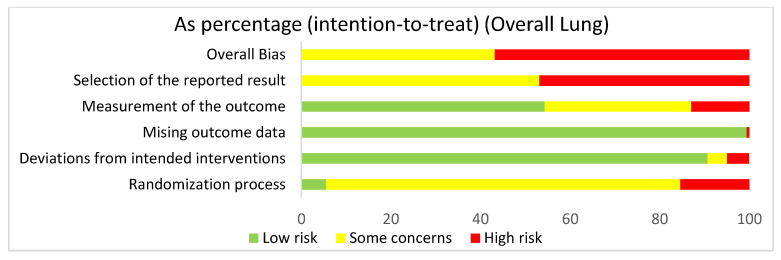
The overall risk of bias for all of the lung studies was a high risk of bias (56.8%) and some concerns (43.2%). The selection of the reported result had a high risk of bias (46.9%) and some concerns (53.1%). The measurement of the outcome had a high risk of bias (13%), some concerns (32.7%), and low risk of bias (54.3%). The missing outcome data domain had a low risk of bias (99.4%) and a high risk of bias (0.6%). Deviations from intended interventions had a low risk of bias (90.7%), some concerns (4.3%), and high risk of bias (4.9%). The randomization process had a high risk of bias (15.4%), some concerns (79%), and low risk of bias (5.6%).

In assessing the overall risk of bias within mouse and human studies that focused on the lung, 56.8% of studies demonstrated high risk of bias, while 43.2% of studies demonstrated some concerns (Figure 3). The greatest contributor to bias in these studies seemed to be the selection of the reported result, which yielded some concerns (53.1%) and had a high risk of bias (46.9%). The missing outcomes domain and deviations from intended interventions domain yielded mostly a low risk of bias of 99.5% and 90.7%, respectively. The measurement of the outcome domain yielded low risk of bias (54.3%) and some concerns (32.7%). The randomization process had some concerns (79%) and high risk of bias (15.4%). Bias may have been introduced in the published literature because most studies were done in mice. Furthermore, since none of the studies were randomized control trials, none of the studies had preregistered protocols, leading to significant biases according to the Cochrane RoB 2.0 tool. When comparing the overall risk of bias for the lung to the overall risk of bias data for both the lung and the liver, the domains seem to be similar, supporting the fact that there were more studies using the lung than the liver.

#### 3.4.3. Lung: Human Studies Risk of Bias Assessment

**Figure 4:**
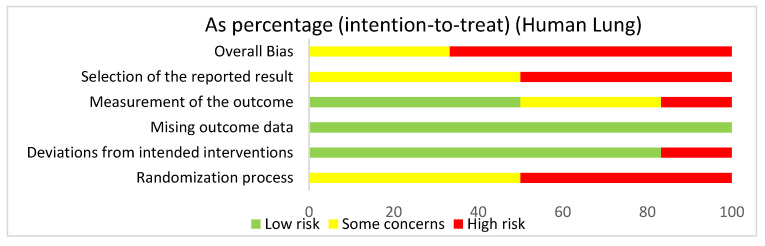
The overall bias for studies regarding the lung utilizing human subjects was a high risk of bias (66.7%) and some concerns (33.3%). The selection of the reported result had a high risk of bias (50%) and some concerns (50%). The measurement of the outcome had a high risk of bias (16.7%), some concerns (33.3%), and low risk of bias (50%). The missing outcome data domain had a low risk of bias of 100%. Deviations from intended interventions had a low risk of bias (83.3%) and a high risk of bias (16.7%). The randomization process had a high risk of bias (50%) and some concerns (50%).

In assessing overall risk of bias within human studies that focused on the lung, 66.7% of studies demonstrated a high risk of bias, while 33.3% of studies demonstrated some concerns (Figure 4). The greatest contributor to bias in these studies seemed to be the randomization process and the selection of the reported result, which both had a high risk of bias (50%) and some concerns (50%). The deviations from intended interventions domain yielded a mostly low risk of bias of 83.3%. The measurement of the outcome domain yielded a low risk of bias (50%) and some concerns (33.3%). The missing outcome data domain had a 100% low risk of bias. Bias may have been introduced in the published literature because most of the human studies did not have information about the randomization or blinding processes used during randomization.

#### 3.4.4. Lung: Mouse Studies Risk of Bias Assessment

**Figure 5:**
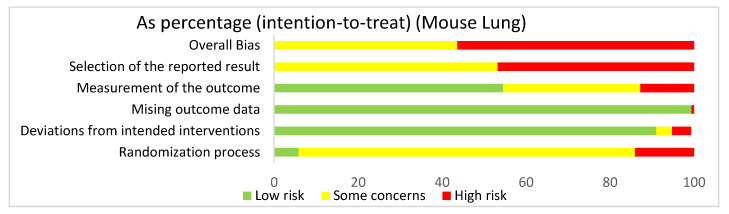
The overall bias for the studies regarding the lung utilizing mouse models was a high risk of bias (56.4%) and some concerns (43.6%). The selection of the reported result had a high risk of bias (46.8%) and some concerns (53.2%). The measurement of the outcome had a high risk of bias (12.8%), some concerns (32.7%), and low risk of bias (54.5%). The missing outcome data domain had a low risk of bias (99.4%) and a high risk of bias (0.6%). Deviations from intended interventions had a low risk of bias (91%), some concerns (3.8%), and high risk of bias (4.5%). The randomization process had a high risk of bias (14.1%), some concerns (80.1%), and low risk of bias (5.8%).

In assessing overall risk of bias within mouse studies that focused on the lung, 56.4% of studies demonstrated a high risk of bias, while 43.6% of studies demonstrated some concerns (Figure 5). The greatest contributor to bias in these studies seemed to be the selection of the reported result domain, which had some concerns (53.2%) and a high risk of bias (46.8%). The missing outcome data domain and deviations from intended interventions domain yielded mostly low risks of bias of 99.4% and 91%, respectively. The measurement of the outcome domain yielded low risk of bias (54.5%) and some concerns (32.7%). The randomization process yielded some concerns (80.1%) and high risk of bias (14.1%). Bias may have been introduced in the published literature because these studies utilized mice. Furthermore, since these studies were not randomized control trials, they did not include preregistered protocols and had multiple eligible analyses, leading to high risk of bias in the selection of the reported result domain.

#### 3.4.5. Liver: Overall Risk of Bias Assessment

**Figure 6:**
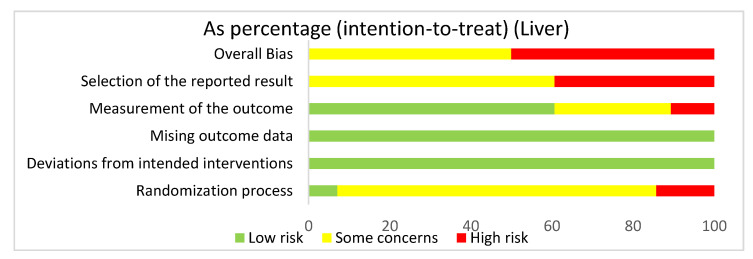
The overall bias for all the studies regarding the liver was a high risk of bias (50%) and some concerns (50%). The selection of the reported result had a high risk of bias (39.3%) and some concerns (60.7%). The measurement of the outcome had a low risk of bias (60.7%), some concerns (28.6%), and high risk of bias (10.7%). The missing outcome data and the deviations from intended interventions domain had a low risk of bias of 100%. The randomization process had a high risk of bias (14.3%), some concerns (78.6%), and low risk of bias (7.1%).

In assessing overall risk of bias within mouse studies that focused on the liver, 50% of studies demonstrated some concerns, while 50% of studies demonstrated a high risk of bias (Figure 6). The greatest contributor to bias in these studies seemed to be the selection of the reported result, which had some concerns (60.7%) and a high risk of bias (39.3%). The measurement of the outcome domain yielded low risk of bias (60.7%) and some concerns (28.6%). The randomization process had some concerns (78.6%) and low risk of bias (7.1%). The missing outcome data domain and the deviations from intended interventions domain both had a low risk of bias of 100%. Bias may have been introduced in the published literature because all studies were done in mice. Furthermore, none of the studies had preregistered protocols and multiple eligible analyses, leading to significant biases.

#### 3.4.6. Summary of Risk of Bias Assessments

The risk of bias assessments revealed that the highest risk of bias domains were selection of the reported result and the randomization process. Our assessments using PRISMA guidelines thus highlight the need for randomized clinical trials in order to improve our understanding of the role of HIMF. As an example, roles for HIMF in the lung could either parallel or diverge from those in the liver. Moreover, in the liver, the majority of studies highlighted were models of acute liver injury, where the acute process could differ in pathogenesis from the chronic injury process.

## 4. Discussion

**Figure 7:**
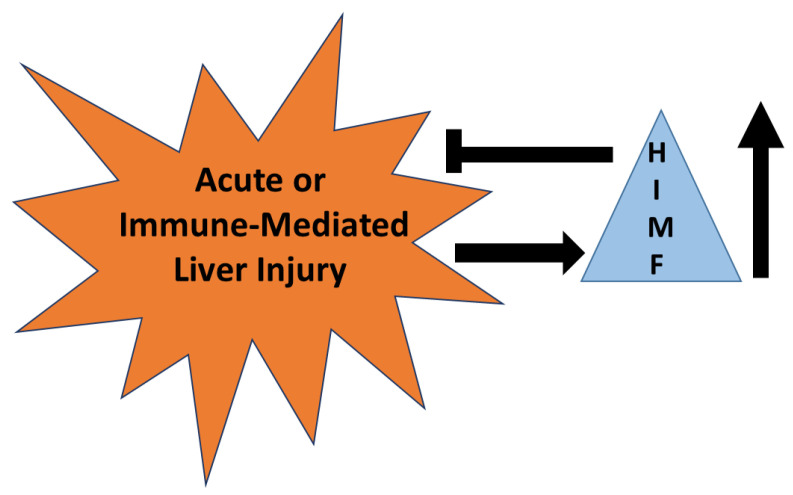
The role of HIMF in acute or immune-mediated liver injury. HIMF is upregulated (↑) in acute or immune-mediated liver injury. Once up-regulated, HIMF may have a role in reducing the severity of injury that occurs.

HIMF has been established as a marker for alternatively activated macrophages that are known to reduce acute liver injury in some models [89,90]. HIMF is upregulated in murine models of acute liver injury and acetaminophen intoxication and is required for the reduction of injury in these models. Even so, there are no studies defining HIMF as a marker of injury or repair in liver injury induced by drugs. Mechanisms described for HIMF in the lung demonstrate significant roles for IL-4, IL-13, and STAT6, all mechanisms known to initiate immune-mediated liver injury. These parallels suggest to us that HIMF is likely upregulated in immune-mediated liver injury and likely downregulates this form of injury (Figure 7). However, caution must be observed since HIMF promotes pulmonary fibrosis; hence, HIMF may have pathogenic roles in chronic forms of liver injury that result in fibrosis.

In conclusion, there are no papers that discuss the role of HIMF in drug-induced or any form of immune-mediated liver injury in humans, even though the evidence for the protection by HIMF in reliable mouse models of immune-mediated liver injury is quite compelling. Drug-induced immune-mediated liver injury is a significant health-related issue. Recent updates in RUCAM (Roussel Uclaf Causality Assessment Method) have provided tools that can be utilized to prospectively assess patients with drug-induced immune-mediated liver injury [91]. Further investigations are necessary in order to determine whether HIMF can be utilized as a marker or therapeutic target in acute or immune-mediated liver injury along with the revised RUCAM update. These studies might provide additional support that could improve the understanding of the molecular basis of this disease.

## Figures and Tables

**Table 1:** Detailed keywords used in search strategy.

Population	Intervention	Comparison Intervention	Outcome Measure
Mouse Mice Rat Squirrel Hamster Guinea Pig Rodent Human Patient BALB/C C57BL/6 Knockout	Resistin-like molecules HIMF RELM-a FIZZ1 Retnla RELM-beta	Liver Lungs	Proinflammatory Inflammatory Protection Pathogenesis

HIMF, Hypoxia-induced mitogenic factor (HIMF), RELM-α, resistin-like molecule α, FIZZ1, found in inflammatory zone 1, retlna, resistin-like alpha, RELM, resistin-like molecule.

## Data Availability

Data is contained within the article.
